# Pricing, management and decision-making of financial markets with artificial intelligence: introduction to the issue

**DOI:** 10.1186/s40854-021-00302-9

**Published:** 2021-11-04

**Authors:** Feng Xiao, Jintao Ke

**Affiliations:** 1 School of Business Administration, Faculty of Business Administration, Southwestern University of Finance and Economics, Chengdu, China; 2 Department of Civil Engineering, The University of Hong Kong, Hong Kong, China

While the advancement of Internet Technology generates massive amounts of data that can facilitate decision makings of financial markets, it also arouses new challenges to financial activities, such as the acquisition, processing and analysis of multiple information resources. In addition, the external environment of the financial market is full of uncertainties, such as the occurrence of COVID-19 pandemic and government intervention, which makes the asset pricing and management much more challenging. In recent years, artificial intelligence technology has developed rapidly and has been widely used in various fields, including the financial markets. Due to its capability of mining patterns from big data, artificial intelligence is regarded as an efficient tool to address the above-mentioned challenges. Methods such as deep learning and reinforcement learning can effectively capture the nonlinear and complex relationship among different financial factors from the big data and support the decision makings of different financial tasks. In this special issue, we provide an overview of the latest applications of deep learning, reinforcement learning, fuzzy theory and other artificial intelligence methodologies on pricing, management, and decision-making of financial markets. The papers in this special issue show that the adoption of artificial intelligence technologies in finance can help us obtain more accurate and robust results and make better decisions.

This volume is the 28th issue of Financial Innovation (FIN), Volume 8, No. 4 (2021). In this issue, scholars from Brazil, China, Italy, Thailand, Turkey have contributed their most up-to-date studies mainly on the new technologies used in the financial markets. The topics of the 15 collected papers include, but are not limited to, the following aspects of artificial intelligence for financial markets:

- Price prediction (4 papers)

Artificial intelligence models, including deep learning and reinforcement learning, have been widely adopted in price prediction and have demonstrated satisfactory results.

The paper “An efficient stock market prediction model using hybrid feature reduction method based on variational autoencoders and recursive feature elimination” predicts the hourly movement directions of eight banking stocks in Borsa Istanbul using historical stock prices and technical indicators as features. It uses linear-based (SVM), deep-learning (LSTM) and ensemble learning (LightGBM) models for the prediction task and compares the model performances in terms of accuracy and F-measure metrics.

The paper “DAViS: a unified solution for data collection, analyzation, and visualization in real-time stock market prediction” proposes a unified solution for data collection, analysis, and visualization in real-time stock market prediction to retrieve and process relevant financial data from news articles, social media, and company technical information. The findings confirm that leveraging an ensemble scheme of machine learning methods with contextual information can improve stock prediction performance.

The paper "Forecasting directional movement of Forex data using LSTM with technical and macroeconomic indicators" applies two separate LSTM models to predict the directional movement of the EUR/USD currency pair, demonstrating satisfactory predictive performance in the experiments based on real forex data.

The paper "Take Bitcoin into your portfolio: A novel ensemble portfolio optimization framework for broad commodity assets" proposes a novel ensemble portfolio optimization (NEPO) framework utilized for broad commodity assets, which integrates a hybrid variational mode decomposition-bidirectional long short-term memory deep learning model for future returns forecast and a reinforcement learning-based model for optimizing the asset weight allocation. The empirical results indicate that the NEPO framework effectively improves prediction accuracy and demonstrates strong trend prediction ability across various commodity assets from different sectors.

- Financial risk management (4 papers)

Financial risk management is an important component of the financial markets and has attracted extensive attentions from both the academic and industry. According to the different research objects, we divide the studies into three major categories.

The first category focuses on the risk management of customers who use financial services. The paper "A high-dimensionality-trait-driven learning paradigm for high dimensional credit classification" proposes a high-dimensionality-trait-driven learning paradigm to conduct feature extraction and classifier selection. The results identify the situation at which using PCA and a single linear classifier is better, and the situation at which the adoption of nonfeature extraction linear ensemble classifier is more effective.

The second category stands from the viewpoint of platforms that provide financial services. Based on regression, the paper "Fintech platforms: Lax or careful borrowers’ screening?" constructs a new proxy to investigate lending platform misconduct, compare the FICO score and LendingClub credit grade, and provides valuable insights to policymakers and lending platforms. The paper "To supervise or to self-supervise a machine learning based comparison on credit supervision" investigates the need for credit supervision conducted by on-site banking supervisors. The findings of machine learning classifier show that the overall performance of the on-site supervision approach is consistently higher than that of the self-supervision approach, justifying the need for on-site credit portfolio examination conducted by the Central Bank.

The third category is from the perspectives of third-party rating agencies. The paper "Detecting conflicts of interest in credit rating changes: a distribution dynamics approach" uses a novel distribution dynamics approach to compare the adjustments of credit ratings by an investor-paid credit rating agency (CRA) and an issuer-paid CRA. It is found that investor-paid ratings are more likely to be downgraded than issuer-paid ratings only in the lower rating grades, whereas issuer-paid CRAs are more concerned with providing accurate ratings.

- Decision making (7 papers)

Decision-making is an essential component of financial markets. The studies have employed fuzzy theory and machine learning approaches to conduct influencing factor analysis and text processing, which help enhance decision making in financial markets.

Based on the fuzzy set theory, the paper "A hybrid heterogeneous Pythagorean fuzzy group decision modelling for crowdfunding development process pathways of fintech-based clean energy investment projects" evaluates the crowdfunding alternatives regarding new service development process pathways of clean energy investment projects; the paper "Consensus-based multidimensional due diligence of fintech-enhanced green energy investment projects" provides a hybrid group decision making approach to assess fintech-based financial alternatives in the green energy investment projects; the paper "Fintech investments in European banks: a hybrid IT2 fuzzy multidimensional decision-making approach" evaluates Fintech-based investments of European banking service; the paper "Identifying the key factors of subsidiary supervision and management using an innovative hybrid architecture in a big data environment" analyzes the dependency and feedback relations among variables of parent/subsidiary gaps and conflicts.

Based on machine learning, the paper "How text sentiment moderates the impact of motivational cues on crowdfunding campaigns" focuses on the moderating effect of text sentiment on the motivational cues in crowdfunding campaigns; the paper " Machine learning approach to drivers of bank lending: evidence from an emerging economy" proposes various algorithms that determine drivers of bank lending; the paper " Recent innovation in benchmark rates (BMR): evidence from influential factors on Turkish Lira Overnight Reference Interest Rate with machine learning algorithm" examines the determinants of Turkish Lira Overnight Reference Interest Rate (TLREF).

